# Supplementary material for: Microbiota Community Structure and Interaction Networks within Dermacentor silvarum, Ixodes persulcatus, and Haemaphysalis concinna
Source: Animals (Basel). 2022 Nov 22;12(23):3237. doi: 10.3390/ani12233237 (PMC9735619; doi:10.3390/ani12233237)
Supplement: Supplementary file 1 [file animals-12-03237-s001.zip › animals-1997223-supplementary.pdf]

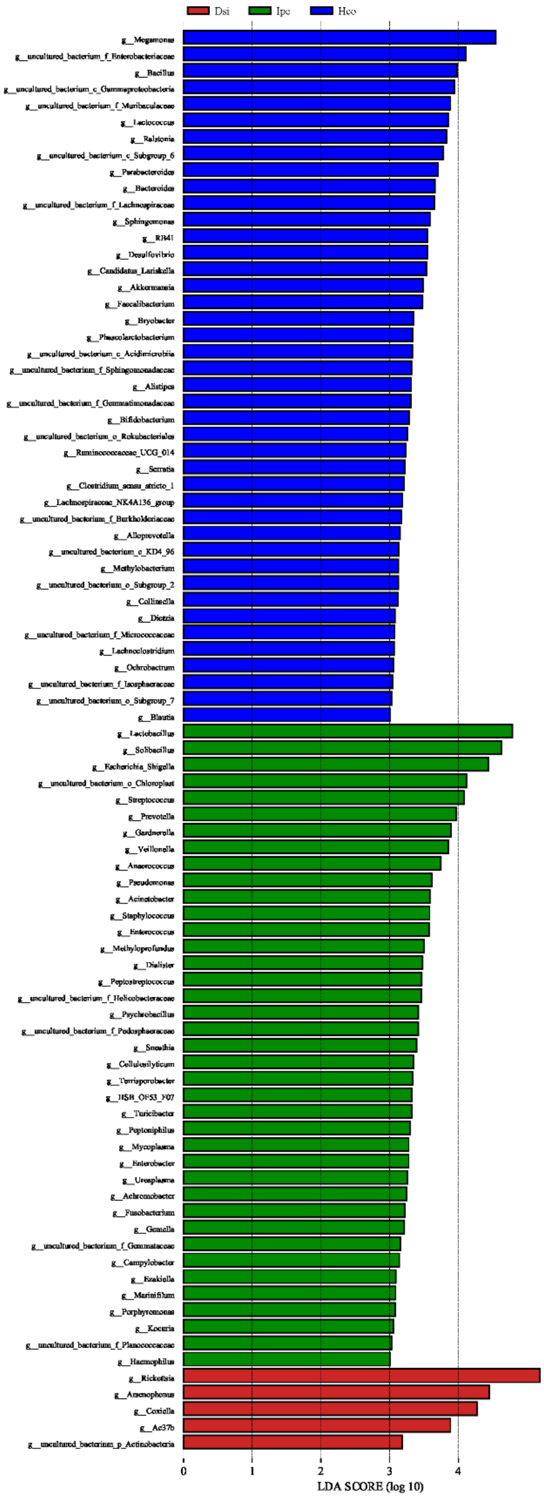

FIGURE S1. LEfSe conducted based on bacterial community in unfed female ticks.

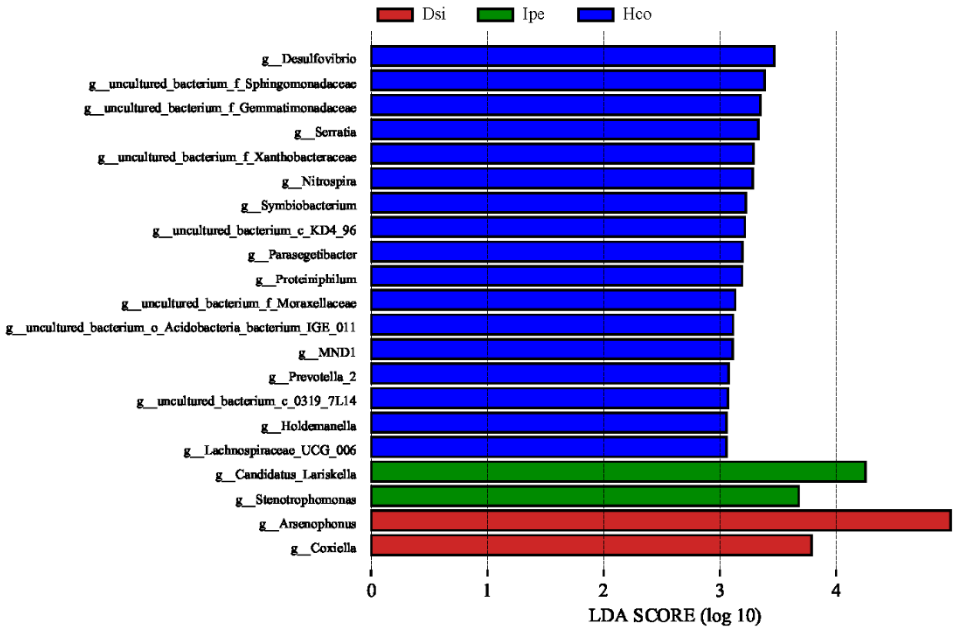

FIGURE S2. LEfSe conducted based on bacterial community in engorged female ticks.

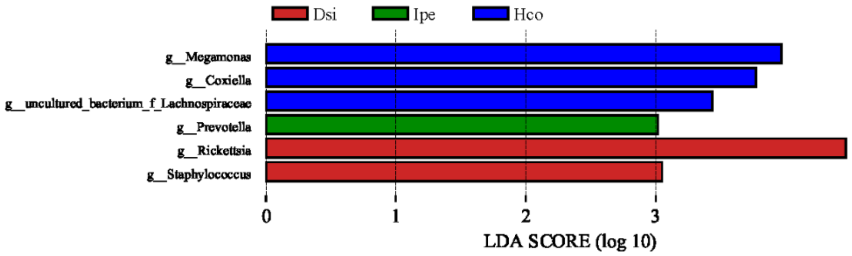

FIGURE S3. LEfSe conducted based on bacterial community in male ticks.

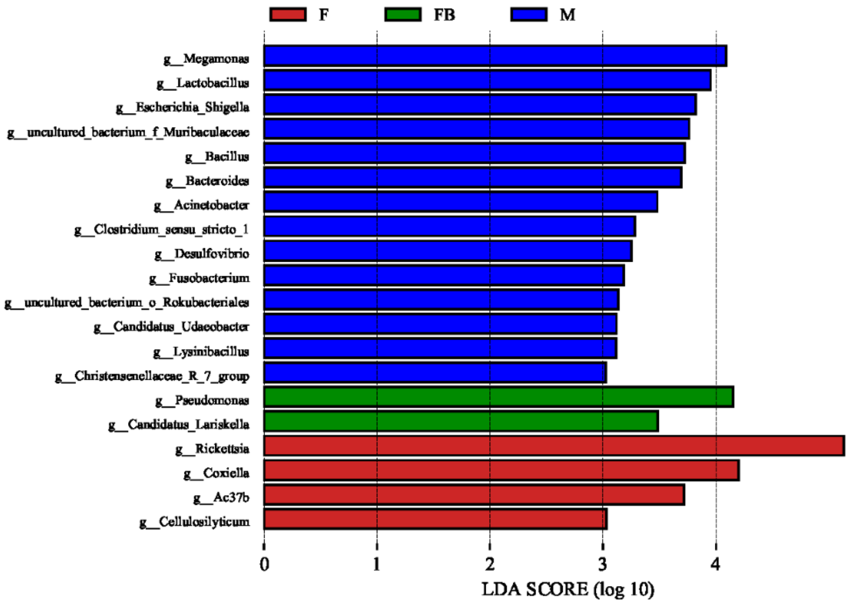

FIGURE S4. LEfSe conducted based on bacterial community in *Dermacentor silvarum*.

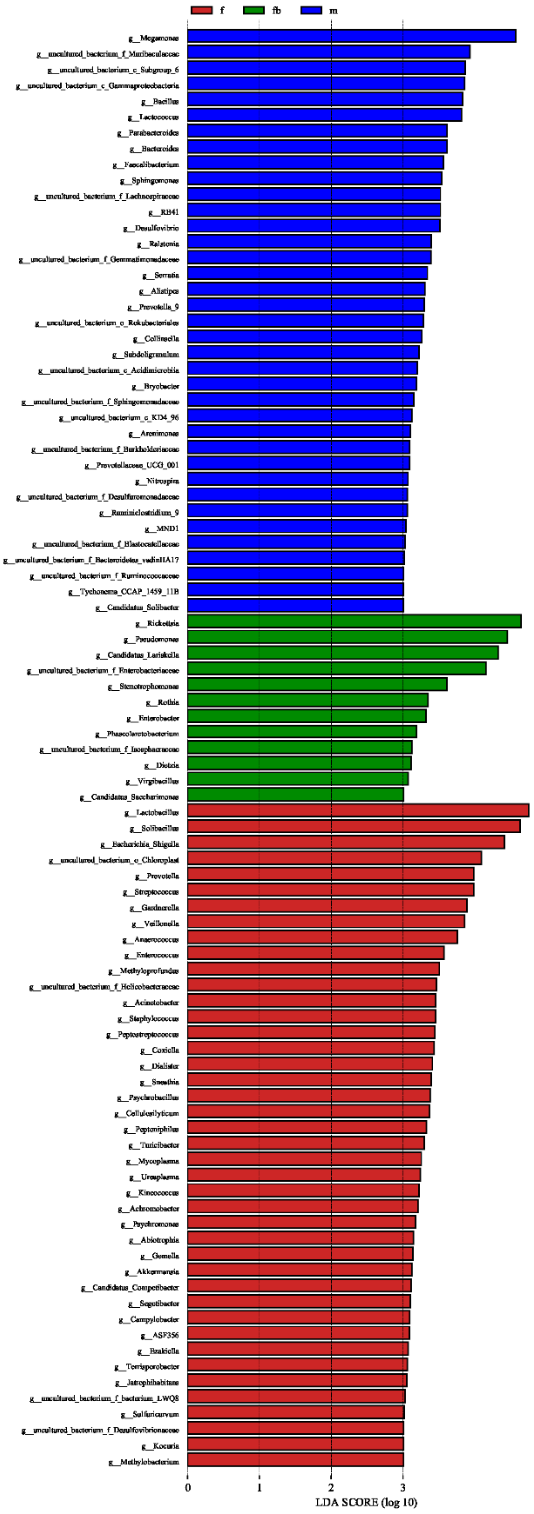

FIGURE S5. LEfSe conducted based on bacterial community in *Ixode spersulcatus*

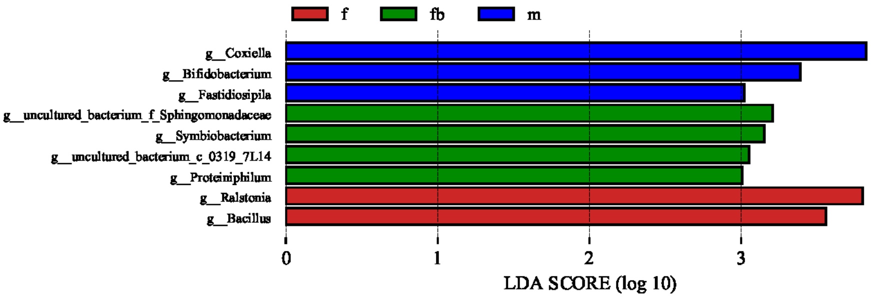

FIGURE S6. LEfSe conducted based on bacterial community in *Haemaphysalis concinna*

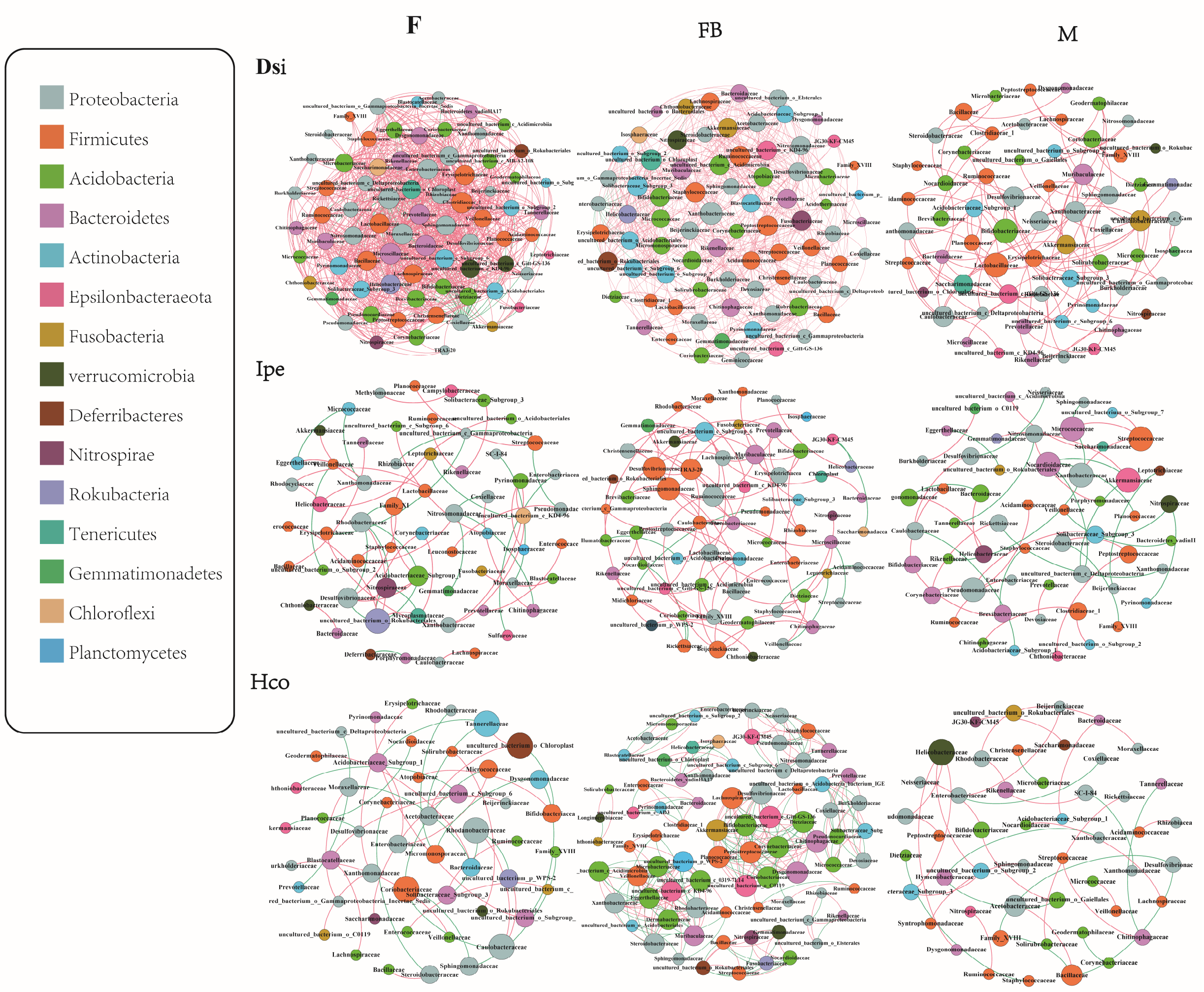

Figure S7. Microbial interaction networks within ticks
